# Role of P2Y Receptors in Platelet Extracellular Vesicle Release

**DOI:** 10.3390/ijms21176065

**Published:** 2020-08-23

**Authors:** Aleksandra Gąsecka, Sylwester Rogula, Ceren Eyileten, Marek Postuła, Miłosz J. Jaguszewski, Janusz Kochman, Tomasz Mazurek, Rienk Nieuwland, Krzysztof J. Filipiak

**Affiliations:** 1 1st Chair and Department of Cardiology, Medical University of Warsaw, 02-106 Warsaw, Poland; sylwesterrogula@o2.pl (S.R.); jkochman@tlen.pl (J.K.); tmazurek@kardia.edu.pl (T.M.); krzysztof.filipiak@wum.edu.pl (K.J.F.); 2 Laboratory Experimental Clinical Chemistry, and Vesicle Observation Center, Amsterdam UMC, University of Amsterdam, 1012 WX Amsterdam, The Netherlands; 3 Department of Experimental and Clinical Pharmacology, Centre for Preclinical Research and Technology, Medical University of Warsaw, 02-097 Warsaw, Poland; cereneyileten@gmail.com (C.E.); mpostula@wum.edu.pl (M.P.); r.nieuwland@amsterdamumc.nl (R.N.); 4 1st Department of Cardiology, Medical University of Gdansk, 80-210 Gdańsk, Poland; jamilosz@gmail.com

**Keywords:** extracellular vesicles, P2Y1 receptors, P2Y12 receptors, antiplatelet therapy, clopidogrel, ticagrelor

## Abstract

Platelet extracellular vesicles (PEVs) are potential new biomarkers of platelet activation which may allow us to predict and/or diagnose developing coronary thrombosis before myocardial necrosis occurs. The P2Y1 and P2Y12 receptors play a key role in platelet activation and aggregation. Whereas the P2Y1 antagonists are at the preclinical stage, at present, the P2Y12 antagonists are the most effective treatment strategy to prevent stent thrombosis after percutaneous coronary intervention. Despite an increasing number of publications on PEVs, the mechanisms underlying their formation, including the role of purinergic receptors in this process, remain an active research field. Here, we outline the clinical relevance of PEVs in cardiovascular disease, summarize the role and downstream signalling of P2Y receptors in platelet activation, and discuss the available evidence regarding their role in PEV formation.

## 1. Introduction

Platelets are anucleated blood cells that play a critical role in haemostasis through their ability to aggregate and form thrombi to stop bleeding [1]. Under normal conditions, prostaglandin E1 (PGE_1_) released from endothelial cells counteracts platelet adherence to the vascular endothelium [2]. PGE_1_ activates adenylate cyclase (AC), which increases the concentrations of cyclic adenosine monophosphate (cAMP), therefore preventing platelet activation [3]. The constant synthesis of PGE_1_ by healthy endothelium is probably one of the main reasons that platelets do not spontaneously aggregate in vivo [2]. Endothelial injury, for example, by rupture or erosion of the atherosclerotic plaque, abolishes the protective effect of PGE_1_ and leads to platelet activation with subsequent obstruction of the coronary arteries [4].

In the absence of PGE_1_, adenosine diphosphate (ADP) released from activated platelets and erythrocytes is one of the most important platelet agonists. There are two main types of receptors that mediate ADP-induced platelet activation: P2Y1 and P2Y12 receptors [5]. Platelets also express the P2Y13 and P2Y14 receptors, but the contribution of these receptors to platelet functionality has not been established [6,7,8]. In turn, the P2Y13 receptor is mostly involved into cholesterol and glucose metabolism, bone homeostasis, pain transmission, and neuroprotection in central nervous system, whereas its role in platelet activation has not yet been established [6,7]. On the other hand, the P2Y14 receptor mediates vasoconstriction of the smooth muscle cells [9]. The P2Y1 and P2Y12 receptors are purinergic G protein-coupled receptors for ADP that are widely distributed within cells, including platelets [10]. The P2Y1 receptor is involved in initiating platelet activation, whereas the P2Y12 receptor increases the sensitivity of platelet activation by other agonists [5,10]. Due to the key role of P2Y1 and P2Y12 receptors in platelet activation, specific receptor antagonists have been developed to decrease the risk of arterial thrombosis. Whereas P2Y1 antagonists are at the preclinical stage [11], at present, P2Y12 antagonists are the most effective treatment strategy in patients with acute coronary syndrome (ACS) undergoing percutaneous coronary intervention (PCI) with stent implantation [12].

The contribution of platelets to health and disease considerably exceeds their role in haemostatic and thrombotic processes [13]. Activated platelets secrete over 300 active substances from their intracellular granules that are mostly involved in wound cleaning and healing, platelets form conjugates with leukocytes leading to leukocyte activation, and platelets release exosomes and microparticles (also called microvesicles or ectosomes) [14,15].

Because (i) different vesicle types overlap with each other in physical properties and in biochemical composition and (ii) currently there are no criteria to accurately distinguish vesicle types, for the vesicles released from platelets, it is best to use the umbrella term “extracellular vesicles” (EVs), which was introduced in 2011 by the International Society of Extracellular Vesicles to encompass all EVs [14]. Platelet EVs (PEVs) are biomarkers of platelet activation and coronary thrombus formation because PEVs are released from activated platelets and platelet-rich aggregates during early stage thrombosis, which may allow prediction and/or diagnosis of developing coronary thrombosis before the onset of myocardial necrosis or monitoring of the response to treatment with P2Y12 antagonists [16,17]. Besides, EVs may play a role in adverse post-infarct cardiac remodelling, and therefore, PEVs are potential therapeutic targets also in patients after myocardial infarction [18]. Due to the broad potential clinical application of (P)EVs as biomarkers, the number of publications on PEVs is growing [16]. To develop (P)EV-based biomarkers, the mechanisms underlying their formation should be clarified. In this review, we outline the clinical relevance of PEVs in cardiovascular disease, summarize the role and downstream signalling of P2Y receptors in platelet activation, and discuss the available evidence regarding their role in PEV formation.

## 2. Platelet Extracellular Vesicles in Cardiovascular Disease: Diagnosis, Prognosis, and Treatment

**Figure 1:**
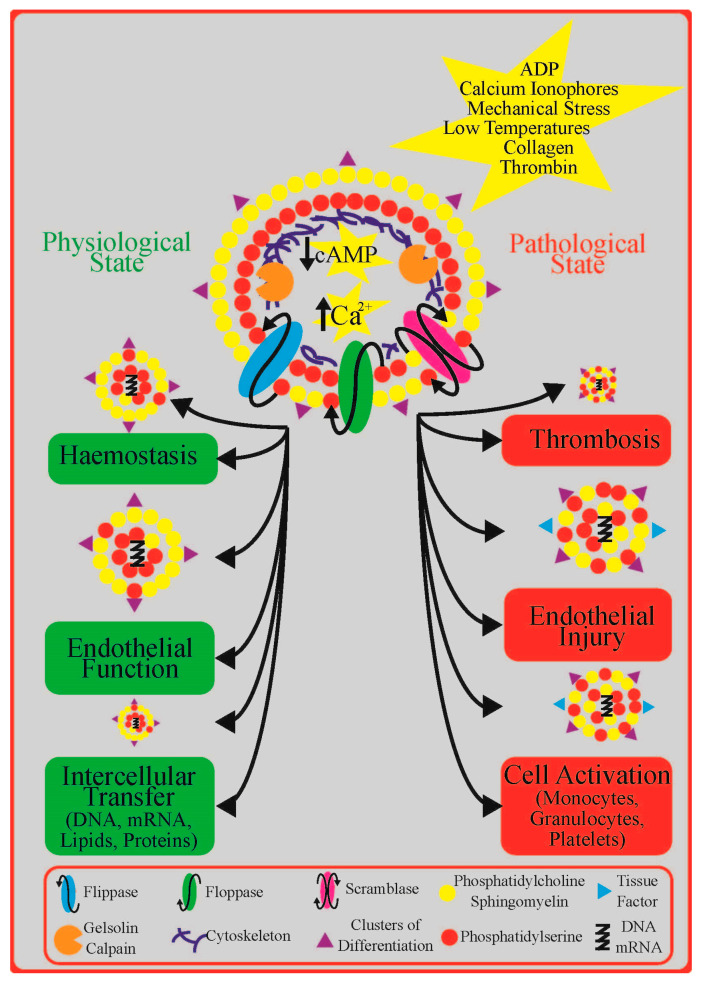
The role of platelet extracellular vesicles (PEVs) in physiological and pathological conditions and the cellular mechanisms underlying their formation: a detailed description can be found in the text. Abbreviations: ADP—adenosine diphosphate; cAMP—cyclic adenosine monophosphate DNA—deoxyribonucleic acid; mRNA—messenger ribonucleic acid; Ca^2+^—calcium.

PEVs (i) are spherical particles surrounded by a phospholipid bilayer (membrane); (ii) have a diameter <1 μm; (iii) retain cytoplasmic components such as proteins, lipids, second messengers, and genetic information; and (iv) expose transmembrane receptors such as integrins derived from the parent cells [19]. Figure 1 shows the role of PEVs in physiological and pathological conditions and the cellular mechanisms underlying their formation. Under steady-state conditions, PEVs are thought to be constitutively released from platelets into their environment, where they serve as important mediators of intercellular communication, contribute to haemostasis, and protect vascular endothelium [19]. In response to platelet activation by ADP or other agonists, shear stress, or low temperature, the release of PEVs increases [19]. Hence, both concentration and functional properties of circulating PEVs change in most if not all pathological conditions associated with abundant platelet activation, including cardiovascular disease, which may be either due to increased formation or decreased clearance [20,21,22]. To which extent the bulk of “PEVs” that are present in the human circulation is derived from platelets or from platelet precursor cells, megakaryocytes, is obscure, but only PEVs originating from platelets are thought to expose specific platelet activation proteins such as P-selectin (CD62P; CD: a cluster of differentiation) [23].

There is evidence that PEVs may contribute to inflammation, thrombosis, and atherosclerosis because they (i) interact with endothelial cells, thereby promoting endothelial injury; (ii) adhere to leukocytes via receptors such as P-selectin, which leads to production and secretion of cytokines and tissue factor (TF); and (iii) activate resting platelets, which amplifies thrombo-inflammatory responses [14,24]. Moreover, PEVs may be procoagulant due to the exposure of negatively charged phospholipids, which provide a surface for the assembly of blood clotting factors, and perhaps to the still disputed presence of TF, the initiator of coagulation in vivo [24]. Based on these findings, PEVs may (i) provide a new diagnostic tool in cardiovascular diseases [16], (ii) improve the accuracy of current models for cardiovascular risk assessment [22], and (iii) provide a new therapeutic target in the treatment of cardiovascular diseases [18].

## 3. Platelet P2Y Receptors: Role and Downstream Signalling

There are two distinct P2Y receptors on platelets that bind ADP: the P2Y1 receptor, which is involved in platelet activation, and the P2Y12 receptor, which increases the sensitivity of platelets to stimulation [5,10]. Activation of P2Y1 results in a weak and transient aggregation of platelets in vitro [5]. However, the P2Y1 receptor seems nevertheless indispensable in in vivo thrombus formation because, in mouse models, both pharmacological inhibition and genetic deficiency are associated with resistance to thrombosis [25] whereas overexpression was found to enhance thrombosis [26]. The P2Y12 receptor on the other hand has a completely different mode of action. Activation of this receptor makes platelets more susceptible and sensitive to activation by platelet agonists other than ADP by decreasing the cytosolic concentration of cAMP [5]. Due to its key role in the growth and stabilization of a thrombus as well as due to its restricted tissue distribution, the P2Y12 is an established target for antithrombotic drugs [12], although one has to keep in mind that both P2Y1 and P2Y12 receptors must act in concert to achieve an appropriate platelet aggregation and that inhibition of intracellular signalling through either of the P2Y receptors blocks ADP-induced platelet activation [3].

**Figure 2:**
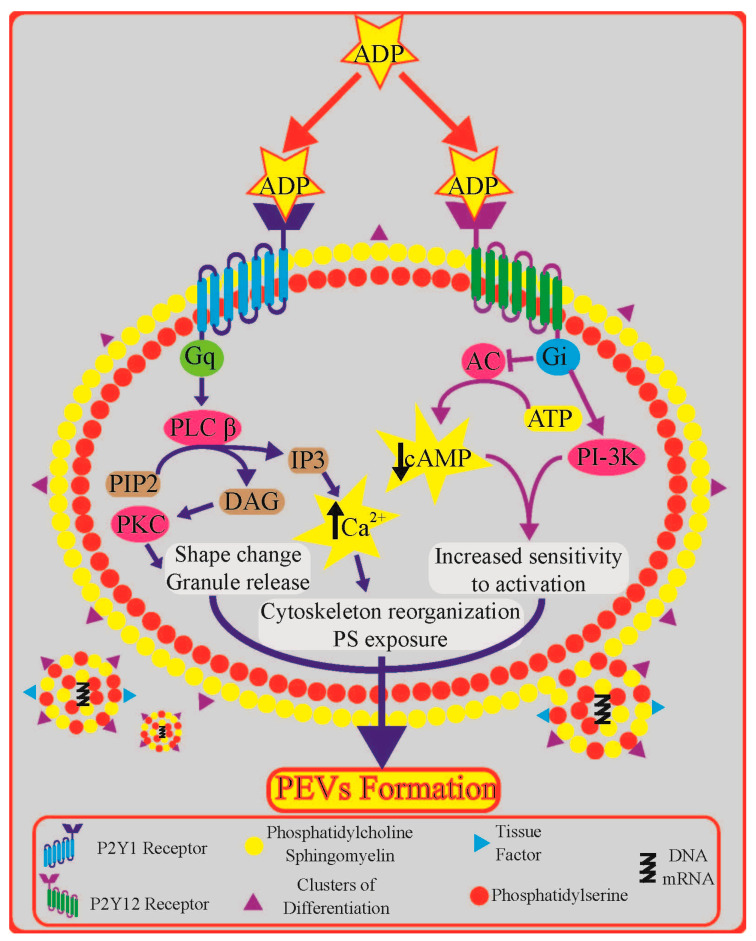
The P2Y1- and P2Y12-mediated signal transduction pathway and their potential involvement in platelet extracellular vesicles (PEVs) formation: a detailed description can be found in the text. Abbreviations: AC—adenylyl cyclase; ADP—adenosine diphosphate; ATP—adenosine triphosphate; cAMP—cyclic adenosine monophosphate; Ca^2+^—calcium; DAG—diacylglycerol; Gi—GTP-binding protein i; Gq—GTP-binding protein q; IP3—inositol trisphosphate; PIP2—phosphatidylinositol bisphosphate; PI3-K—phosphoinositide 3-kinase; PKC—protein kinase C; PLCβ—phospholipase C β.

The P2Y1- and P2Y12-mediated signal transduction pathways and their involvement in PEV formation are showed in Figure 2. Binding of ADP to the Gq (GTP-binding protein q)-coupled P2Y1 receptor activates phospholipase C (PLC) β. In turn, PLCβ hydrolyses membrane phosphatidylinositol bisphosphate to inositol trisphosphate and diacylglycerol [27]. This signalling cascade leads to an increase in the intracellular Ca^2+^ concentration and activation of protein kinase C, together inducing platelet shape change, the release of granule contents, and aggregation [27]. Binding of ADP to the Gi-coupled P2Y12 activates two major biochemical pathways: inhibition of cAMP production and activation of phosphoinositide 3-kinase (PI3-K), which phosphorylates various target proteins [3]. Particularly, the decrease in the concentration of cytosolic cAMP is essential because high concentrations of cytosolic cAMP (for example induced by PGI_2_) prevent platelet activation [3]. By decreasing the concentration of cAMP, platelets become more sensitive to activation by any platelet agonist.

## 4. Role of Platelet P2Y Receptors in Platelet Extracellular Vesicle Formation

At present, there is consensus that the formation of PEVs depends on the (i) intracellular calcium (Ca^2+^) concentration, (ii) intracellular cAMP concentration, and (iii) state of phosphorylation of platelet intracellular components [27,28,29]. All second messengers involved in PEV formation are affected by P2Y receptors [27,28,29].

### 4.1. Insights from Signal Transduction Pathways

Platelets release vesicles during aging in response to physiological agonists (e.g., ADP, thrombin, and collagen), compounds that have a direct influence on second messenger levels, calcium ionophores, mechanical stress, or low temperatures [27,29]. Activation of the P2Y1 receptor leads to an increase in cytosolic Ca^2+^ concentration, which is a key step in PEV formation [27]. An elevated cytosolic Ca^2+^ concentration affects the activity of transmembrane inward and outward phospholipid transporters, including flippase, floppase, and scramblase, which together are responsible for maintaining the asymmetric distribution of phospholipids within the phospholipid bilayer of the cell membrane [22]. Under resting conditions, the outer leaflet contains phosphatidylcholine and sphingomyelin whereas the inner layer contains phosphatidylethanolamine and phosphatidylserine (PS). A rapid increase in floppase activity with a decline in flippase activity leads to specific translocation of PS from the inner to outer leaflets, creating transient mass imbalance between the layers [24]. In addition, activation of scramblase results in bidirectional, nonspecific transport of lipids between both leaflets, which further favours their equal distribution [22]. Concurrently, calpain and other Ca^2+^-dependant enzymes degrade the proteins of cell cytoskeleton, which facilitates budding of outer platelet membrane and shedding of EVs [24,29].

One has to bear in mind, however, that in light of recent findings, the presence and amount of PS on PEV surfaces seems questionable because a substantial portion of PEVs presents PS negative [25] and because PS exposure is highly dependent on pre-analytical conditions (collection, handling, and storage of the sample), being rather an in vitro-induced artefact than reflecting in vivo PEV procoagulant activity [25]. Also, the respective involvement of flippase, floppase, scramblase, and calpain in the process of PEV formation is not entirely clear. There is evidence that PEVs can be generated also without transmembrane flip-flop PS movement and without direct calpain activation [22].

The P2Y12-induced decrease in intracellular cAMP concentration increases platelet sensitivity to activation and in parallel promotes PEVs formation [18]. Compounds that increase the intracellular cAMP concentrations or inhibit PI3-K reduce the rate of platelet vesiculation in response to ADP [18]. A similar effect was observed in platelets pre-treated with the cAMP-elevating agent forskolin, which abolished PS exposure and PEV formation in response to thrombin and collagen [24]. These results indicate that PEV formation is partially inhibited under conditions of elevated cytosolic concentration of cAMP and is affected by cAMP-dependent protein kinases [27,28,29]. P2Y12 antagonists (clopidogrel and AR-C69931MX) also interact with prostaglandin E1 (PGE1) to counteract platelet activation under basal conditions by interfering with Ca^2+^ mobilization and by increasing the concentrations of cAMP within the platelet [25].

### 4.2. Insights from Experimental Studies

Following binding of ADP to the P2Y1 and P2Y12 receptors, ADP triggers an increase in cytoplasmic Ca^2+^ concentration and a decrease in cytoplasmic cAMP concentration, respectively, thus promoting platelet activation including the formation of PEVs. However, the roles of these two P2Y receptors in PEV release seem to differ [30]. Thus far, the relative contribution of the P2Y1 and P2Y12 receptors to the release of PEVs was investigated in a few studies only. In our experiments, we preincubated platelet-rich plasma for 30 min in room temperature with saline, the P2Y1 receptor antagonist MRS2179, the P2Y12 receptor antagonist ticagrelor, or a combination of both antagonists (final concentrations 100 and 1 μM, respectively). We found out that ADP-induced platelet aggregation was inhibited by MRS2179 (75% decrease), by ticagrelor (90% decrease), and by a combination of both antagonists (90% decrease). The release of PEV-exposing CD61 was observed already after 30 min, and this release was not significantly inhibited by MRS2179 (34% decrease) or by ticagrelor (37% decrease) but was abolished when both ADP receptors were blocked (66% decrease). On the contrary, the release of PEV-exposing CD61, P-selectin, and PS was observed only after 1 h and this release was not inhibited by MRS2179 (38% decrease) but was decreased by ticagrelor (68% decrease) and by a combination of both antagonists (76% decrease) [29]. Hence, it seems that ADP-activated platelets release two distinct subpopulations of PEV: a fast-formed population of PEV-exposing CD61 but not P-selectin or PS, and a slow-formed population exposing CD61, P-selectin, and PS. The release of these subpopulations differs in their sensitivity to inhibition of different ADP receptors. Whereas the release of CD61+ PEV requires inhibition of both ADP receptors, the release of CD61+/CD62P+/PS+ PEV is sensitive to inhibition of the P2Y12 receptor alone. Because CD62P+/PS+ PEVs are involved in inflammation and thrombosis, the anti-inflammatory effects of the P2Y12 antagonist ticagrelor may in part be due to inhibition of this PEV subpopulation [31]. However, in another study, inhibition of P2Y12 receptor inhibited the release of PEV in response to ADP and other agonists whereas inhibition of the P2Y1 receptor inhibited the release of PEVs only upon activation by ADP [32]. Other authors reported that inhibition of both the P2Y1 and P2Y12 receptors decreases the release of PEVs in response to a combination of ADP and collagen [33]. Altogether, at present, both the P2Y1 and P2Y12 receptors seem to play a role in the release of PEVs although their relative contribution remains to be elucidated.

The different roles of P2Y1 and P2Y12 receptors in PEV release may be because platelet activation by platelet agonists, such as thrombin or collagen, is amplified by ADP-binding to P2Y12. If the binding of ADP to the P2Y12 receptor is inhibited by P2Y12 antagonists, the cAMP level remains high and platelet sensitivity to activation stays low, irrespective of the agonist [33]. On the contrary, inhibition of the P2Y1 receptor blocks only ADP-induced increase in platelet Ca^2+^ concentration whereas the P2Y12 receptor-mediated (and also ADP-induced) decrease in cAMP level is preserved. Consequently, cytosolic cAMP concentration is low and platelets remain sensitive to activation by other platelet agonists [33]. Finally, it is possible that the potency and concentration of the agonist, for example, shear stress versus activation by soluble agonists in vitro, induces the release of different subpopulations of PEVs. At present, how the specific agonists affect PEV formation is not well understood.

### 4.3. Insights from Antiplatelet Therapy

The pleiotropic properties of P2Y12 inhibitors have been demonstrated in numerous studies and are summarized elsewhere [34,35,36,37,38]. These pleiotropic effects may be at least partly due to inhibition of PEV release. The effects of clinically applicable P2Y12 antagonists on (P)EV release are summarized in Table 1. For example, clopidogrel decreased thrombin receptor-activating peptide (TRAP)-induced release of PEVs in patients with acute coronary syndrome [39]. In vitro, where complete P2Y12 receptor inhibition can be achieved, this effect was even more pronounced [40]. Similarly, the active metabolite R-138727 of prasugrel reduced the collagen- and TRAP-induced release of PEV dose dependently in vitro. R-138727 also completely inhibited agonist-induced Ca^2+^ mobilisation in platelets [40], which is not due to blocking the binding of ADP to P2Y1 receptors because the P2Y1 receptor itself is unaffected by P2Y12 inhibitors [41]. The mechanisms beyond inhibition of P2Y1 and P2Y12 receptors which mediate the effect of R-138727 on platelets are not yet elucidated.

When the P2Y12 receptor is completely blocked by R-138727, R-138727 partially inhibits platelet aggregation induced by TRAP and collagen [40]. This observation clearly demonstrates that the secretion of ADP, induced by activation with TRAP and collagen, is required to lower the cytosolic concentration of cAMP, which in turn promotes and enables full-blown platelet activation. Thus, the binding of ADP to the P2Y12 receptor is essential for other potent agonists to achieve full-blown platelet activation.

The novel, reversible P2Y12-inhibitor cangrelor also reduced the release of PEVs in response to collagen and TRAP in vitro [43]. A combination of cangrelor and GP IIb/IIIa antagonists abciximab or tirofiban further potentiated this effect [43], which is in accordance with the previous finding that activated GP IIb-IIIa is a prerequisite for the release of PEVs [47]. Since blocking P2Y12 activation only decreases PEV release whereas blocking platelet–platelet interactions via activated GP IIb-IIIa entirely abolishes PEV release, it seems that PEVs are truly formed from platelet-rich thrombi [47].

Short-term treatment with clopidogrel on top of aspirin did not affect the plasma concentrations of PEVs, EVs exposing TF, and endothelial EVs in a randomized, double-blind, placebo-controlled trial including 44 healthy volunteers compared to aspirin only [42]. Furthermore, in a group of 26 patients with stable coronary artery disease (SCAD), no differences in the plasma concentration of PEVs were observed following administration of aspirin and clopidogrel, although there was an inverse relationship between clopidogrel serum levels and plasma concentrations of PEVs [44]. Similarly, neither PEVs nor endothelial EVs were affected by clopidogrel when compared to aspirin therapy in 20 subjects with coronary heart disease [45]. Finally, withdrawal of P2Y12 antagonists ticagrelor, prasugrel, or clopidogrel had no influence on the plasma concentrations of PEVs, endothelial EVs, monocyte EVs, and erythrocyte EVs in 62 patients with SCAD 12 months after PCI and stent implantation [46].

Given previous discrepancies regarding the “anti-EV” effect of P2Y12 antagonists, we conducted a randomized controlled trial to compare the effect of the new and more potent P2Y12 antagonist ticagrelor and clopidogrel on the release of EVs in 60 patients after acute myocardial infarction (AMI) in a standardized and investigator-blinded way (NCT02931045) [48]. We analysed concentrations of multiple subtypes of EVs. We applied state-of-the-art methods to collect and handle blood samples, calibrated flow cytometry, and automated software to analyse concentrations of EVs [21,48]. We found that ticagrelor attenuates the increase of platelets (CD61^+^ and P-selectin+), fibrinogen+, PS+, and leukocyte EV concentrations 6 months after AMI compared to clopidogrel. We also found a correlation between EVs exposing P-selectin and C-reactive protein and between EVs exposing fibrinogen and platelet reactivity [21]. Whether this effect results from a more potent P2Y12 receptor inhibition by ticagrelor compared to clopidogrel or from other ticagrelor-related off-target effects (for example, inhibition of the reuptake of adenosine) requires further investigation. Nevertheless, because PEVs exposing P-selectin, fibrinogen, and PS are thought to disseminate thrombosis and inflammation, their ongoing release despite treatment with clopidogrel or ticagrelor after AMI (i) may indicate that alternative pathways are involved in PEV generation in vivo, including P2Y1-mediated pathway, and (ii) may explain recurrent thrombotic events despite antiplatelet therapy as well as worse clinical outcomes on clopidogrel compared to ticagrelor [49].

Altogether, it seems that P2Y12 antagonists suppress the release of PEVs in an acute setting and that the more potent P2Y12 antagonists may have a more efficient “anti-EV” effect compared to the less potent antagonists. Further studies are needed to establish whether there is an association between concentrations of EVs and recurrent thrombotic events during treatment with P2Y12 antagonists. As P2Y12 antagonists reduce the prevalence of recurrent thrombotic events in patients with ACS, understanding their contribution to the release of PEVs might result in prominent clinical benefits. P-selectin-exposing PEVs exert pro-inflammatory and prothrombotic effects by activating monocytes and lead to cytokine release and TF exposure on the monocyte surface. In turn, PS-exposing PEVs have prothrombotic effects by providing the binding surface for activated clotting factors. Hence, modulation of the release of different subpopulations of PEVs might be responsible for the combined anti-inflammatory and antithrombotic effects of P2Y12 antagonists [50].

## 5. Other P2Y Receptors: P2Y13 and P2Y14

Although the G-protein-coupled P2Y1 and P2Y12 receptors were shown to play a crucial role in platelet aggregation, therefore becoming a crucial target of antiplatelet therapy, the scientific focus has also recently been placed on other P2Y receptors. The P2Y13 and P2Y14 receptors are recent P2Y receptors identified and novel members of the Gi-coupled P2Y receptor family. Although both the P2Y13 and P2Y14 receptors present platelets, the contribution of these receptors to platelet functionality has not been established [6,7,8]. Therefore, these receptors were initially underestimated. However, recent pharmacologic studies revealed that P2Y13 and P2Y14 may play a role in many pathophysiological processes related to inflammation and thrombosis.

The genes of P2Y12, P2Y13, and P2Y14 receptors reside on chromosome 3 at 3q25.1. The polymorphisms of these genes were reported in humans with yet unknown consequences [6]. The P2Y13 responds to ADP, just like the P2Y12 receptor [6,7]. The canonical signalling of P2Y13 and P2Y14 is coupled to a Gi receptor. However, P2Y13 can also couple to different G proteins (Gs/Gq) and can trigger several intracellular pathways related to the activation of mitogen-activated protein kinases and the phosphatidylinositol 3-kinase [7]. P2Y13 plays a role in the regulation of cholesterol and glucose metabolism, bone homeostasis, and central nervous system function including pain transmission and neuroprotection [49]. For example, P2Y13 has been specifically linked to the modulation of reverse cholesterol transport at the level of hepatocytes [6]. In the study of P2Y13-deficient mice, the activation of P2Y13 receptors was demonstrated to stimulate endocytosis of high-density lipoprotein particles by apolipoprotein A-I [49]. Additionally, in a recent study in P2Y13-deficient mice, osteoclast and osteoblast number were reduced, resulting declined bone turnover. Altogether, the multifaceted and unique features of the P2Y13 receptor may help to clarify their role in pathophysiology, including inflammation and thrombosis.

The presence of the P2Y14 receptors on platelets has recently been documented, with yet unknown functionality [8]. However, the P2Y14 receptors are mostly associated with immune response, inflammation, and endothelial function due to their high expression levels on other various subgroups of leucocytes and epithelial cells. For example, the P2Y14 receptors contribute to chemotaxis in human neutrophils [5]. Historically, the P2Y14 receptors were thought to be strictly uracil-diphosphate (UDP)-glucose-activated, while newer studies have suggested that UDP is also a potent agonist of these receptors [8,9]. At present, there is no evidence that the P2Y14 receptors are activated by ADP [51]. To further study the function of the P2Y14 receptors, the development of P2Y14 antagonists is required. However, because not only UDP-glucose but also other UDP-conjugated sugars have been reported to activate the P2Y14 receptor, it is challenging to develop antagonists which provide complete inhibition of the P2Y14 receptor [50].

Hitherto, not enough scientific attention has been placed on the possible effects of P2Y13 and P2Y14 receptors in the release of PEVs. Since these receptors modulate several intracellular pathways involved in PEV formation, activate various types of protein kinases, and participate especially in the immunity- and inflammation-related processes, their contribution to PEV release cannot be excluded. Furthermore, activation of these receptors could possibly be responsible for the continuous release of certain subpopulations of PEVs despite antiplatelet therapy with the P2Y12 receptor [47]. As new functions of the P2Y13 and P2Y14 receptors also come to light with gene silencing methods, future studies may allow to clarify their involvement in platelet activation and PEV release and may acknowledge their modulation as novel therapeutic targets [49].

## 6. Conclusions and Future Directions

Despite nearly 50 years of extensive research on PEVs, there is no consensus regarding the respective roles of the P2Y1 and P2Y12 receptors in this process. Based on the insights from signal transduction pathways, it seems that both P2Y1 and P2Y12 receptors may potentially play a role in PEV release because they trigger an increase in cytoplasmic Ca^2+^ concentration and a decrease in cytoplasmic cAMP concentration, respectively, thus promoting platelet activation including the formation of PEVs. Based on the experimental studies, it is possible that the potency and concentration of the agonist induces the release of different subpopulations of PEVs, which in turn may be differentially affected by ADP receptor antagonists. Finally, the results of the clinical studies indicate that P2Y12 antagonists may suppress PEV release in an acute setting and that more potent P2Y12 antagonists may have a more efficient “anti-EV” effect. However, the anti-EV effect of P2Y12 antagonists in healthy volunteers and patients with SCAD is less clear.

Importantly, one should keep in mind that the observed discrepancies between studies are still likely to result from substantial methodological limitations in (P)EV measurements, including (i) the lack of guidelines to study EVs in body fluids, (ii) low sensitivity of EV detection techniques (especially flow cytometry), and (iii) lack of calibrated assays to study EVs, altogether resulting in poor reliability, reproducibility, and comparability of the results. However, due to the joint efforts of international experts gathered in the International Society of Extracellular Vesicles and other societies, great progress has been made to standardise EV measurements in recent years. The main milestones include (i) development of standard operating procedures and guidelines for handling and storage of human body fluids for EV analysis [51,52], (ii) improved sensitivity and specificity of PEV detection by flow cytometry [53,54,55], and (iii) development of platforms for standardised reporting of the results [56,57,58].

Improved isolation and detection techniques along with protocols and guidelines have enabled the reevaluation of many previous concepts on the characteristics and biological functions of PEVs, which has been summarized elsewhere [19]. With these improvements, it is now becoming feasible to study for the very first time (P)EVs in multicentre clinical trials and to answer the crucial research question of whether EVs may be applied as biomarkers of disease.

## Figures and Tables

**Table 1:** The effects of clinically applicable P2Y12 antagonists on extracellular vesicle (EV) release.

Condition	Study Group	Control Group	Sample Size	Effect on EVs	Ref.
Healthy volunteers	Clopidogrel	Aspirin	*n* = 44	~ Platele ~ TF ~ Endothelial	[42]
	Prasugrel	No inhibitor	*n* = 18	↓ Platelet	[40]
	Cangrelor	No inhibitor	Data not provided	↓ Platelet	[43]
SCAD	Clopidogrel	Aspirin	*n* = 26	~ Platelet	[44]
	Clopidogrel	Aspirin	*n* = 20	~ Platelet ~ Endothelial	[45]
ACS	Clopidogrel	Aspirin	*n* = 12	↓ Platelet	[39]
	Ticagrelor	Clopidogrel	*n* = 60	↓ Platelet ↓ Phosphatidylserine^+^ ↓ Fibrinogen^+^ ↓ Leukocyte ~ Endothelial ~ Erythrocyte	[21]
	Ticagrelor, Prasugrel, Clopidogrel	Aspirin	*n* = 62	~ Platelet ~ Endothelial ~ Monocyte ~ Erythrocyte	[46]

ACS—acute coronary syndrome; Ref.—reference; SCAD—stable coronary artery disease; TF—tissue factor; “~” no effect; “↓” decrease.

## Abbreviations

AC	Adenylyl cyclase
ACS	Acute coronary syndromes
ADP	Adenosine diphosphate
AMI	Acute myocardial infarction
ATP	Adenosine triphosphate
cAMP	Cyclic adenosine monophosphate
Ca^2+^	Calcium
CD	Cluster of differentiation
DAG	Diacylglycerol
Gi	GTP-binding protein i
Gq	GTP-binding protein q
IP3	Inositol trisphosphate
PCI	Percutaneous coronary intervention
PIP2	Phosphatidylinositol 4,5-bisphosphate
PI3-K	Phosphoinositide 3-kinase
PEVs	Platelet extracellular vesicles
PKC	Protein kinase C
PLC β	Phospholipase C β
PS	Phosphatidylserine
SCAD	Stable coronary artery disease
TF	Tissue factor
TRAP	Thrombin receptor-activating peptide
